# A fully automated morphological analysis of yeast mitochondria from wide-field fluorescence images

**DOI:** 10.1038/s41598-024-81241-0

**Published:** 2024-12-03

**Authors:** Jana Vojtová, Martin Čapek, Sabrina Willeit, Tomáš Groušl, Věra Chvalová, Eva Kutejová, Vladimír Pevala, Leoš Shivaya Valášek, Mark Rinnerthaler

**Affiliations:** 1 Laboratory of Regulation of Gene Expression, Institute of Microbiology of the Czech Academy of Sciences, Vídeňská 1083, Prague, 14220 Czech Republic; 2 Light Microscopy, Institute of Molecular Genetics of the Czech Academy of Sciences, Prague, 14220 Czech Republic; 3 Laboratory of Biomathematics, Institute of Physiology of the Czech Academy of Sciences, Prague, 14220 Czech Republic; 4 Biosciences and Medical Biology, Paris-Lodron-University Salzburg, Hellbrunnerstrasse 34, Salzburg, 5020 Austria; 5 Laboratory of Cell Signalling, Institute of Microbiology of the Czech Academy of Sciences, Prague, 14220 Czech Republic; 6 Department of Biochemistry and Protein Structure, Institute of Molecular Biology, Slovak Academy of Sciences, Bratislava, 84551 Slovakia

**Keywords:** Yeast, Mitochondria, Deep learning, Oxidative stress, Mmi1, TCTP, Cell biology, Microbiology

## Abstract

Mitochondrial morphology is an important parameter of cellular fitness. Although many approaches are available for assessing mitochondrial morphology in mammalian cells, only a few technically demanding and laborious methods are available for yeast cells. A robust, fully automated and user-friendly approach that would allow (1) segmentation of tubular and spherical mitochondria in the yeast *Saccharomyces cerevisiae* from conventional wide-field fluorescence images and (2) quantitative assessment of mitochondrial morphology is lacking. To address this, we compared Global thresholding segmentation with deep learning MitoSegNet segmentation, which we retrained on yeast cells. The deep learning model outperformed the Global thresholding segmentation. We applied it to segment mitochondria in strain lacking the *MMI1/TMA19* gene encoding an ortholog of the human TCTP protein. Next, we performed a quantitative evaluation of segmented mitochondria by analyses available in ImageJ/Fiji and by MitoA analysis available in the MitoSegNet toolbox. By monitoring a wide range of morphological parameters, we described a novel mitochondrial phenotype of the *mmi1Δ* strain after its exposure to oxidative stress compared to that of the wild-type strain. The retrained deep learning model, all macros applied to run the analyses, as well as the detailed procedure are now available at https://github.com/LMCF-IMG/Morphology_Yeast_Mitochondria.

**Supplementary Information:**

The online version contains supplementary material available at 10.1038/s41598-024-81241-0.

## Introduction

Mitochondria are highly dynamic organelles with many essential functions. In addition to ATP production via oxidative phosphorylation (OXPHOS), they are responsible for the citric acid cycle and signaling via its metabolites (see review^1^), iron metabolism (heme biosynthesis and Fe/S cluster synthesis)^2^, thermoregulation^3^, essential lipid and amino acid metabolic pathways, calcium signaling^4^, innate immune signaling^5^, regulation of cell death^6,7^, and cellular aging^2,8^. Optimal mitochondrial function is reflected in mitochondrial morphology.

In the “normal/physiological state”, wild-type yeast mitochondria form an extensive tubular network close to the plasma membrane^9^. The tubular morphology is maintained by two competing processes: fusion (“sticking” of mitochondria together) and fission (“splitting” of mitochondria)^10^. When fusion predominates, the mitochondrial network elongates. The elongated mitochondrial network allows the exchange of damaged elements, such as mtDNA and proteins, between damaged and healthy mitochondria^10^. This can be beneficial under normal physiological conditions and even under moderate stress. Conversely, when fission predominates, it results in a more fragmented network^11^. Fission can be associated with the induction of apoptosis, decreased cellular respiration, and increased ROS production^12^. It is also involved in autophagy of mitochondria (mitophagy)^13^.

In addition to fusion and fission, complete collapse of the mitochondrial network into the spherical organelles can occur as well. The mitochondrial network collapses during stress, a loss of the mitochondrial membrane potential, apoptosis, and cell aging^2,14–16^. It allows a separation of severely damaged mitochondria, which have no membrane potential or produce too many reactive oxygen species (ROS), from the still functional mitochondrial network and their removal by mitophagy^2,17^. Furthermore, it has recently been reported that spherical mitochondria are fusion-incompetent, immobile, and retained in mother cells, which is a fully reversible process when the stress is relieved^16^. Thus, mitochondrial morphology is an important parameter of cellular fitness and health.

Numerous tools can be used for assessing mammalian mitochondrial morphology, such as well-established Mito Hacker^18^. More, recently tools using deep learning for automated analysis of fluorescence microscopy videos/images, including MitoMo^19^ and MitoSegNet^20^, as well as a deep learning model tailored for electron microscopy image segmentation^21^ have been introduced. However, tools for the morphological analysis of the yeast mitochondria are limited. The MitoGraph version for quantifying the volume the yeast tubular mitochondrial networks is available^22^, however, there is a limited number of morphological output parameters. In addition, the technique is optimized for high-resolution spinning-disk confocal microscopy imaging, which is financially costly. Moreover, manual identification of regions of interest (ROIs) around each cell is needed, which is extremely laborious. Similarly, another method, MitoLoc^23^, which was designed for the quantification of mitochondrial morphology and the mitochondrial membrane potential measurements in single yeast cells, is based on super-resolution microscopy and requires manual identification of ROIs.

In the absence of a simple tool for morphological analysis of mitochondria in yeast, some authors probably resort to displaying microscopy images of the mitochondrial phenotype without a detailed evaluation^8,24,25^. Further, many authors evaluate mitochondrial morphology by categorizing cells into different qualitative groups according to the mitochondrial shape (e.g., nontubular or tubular, spherical, fragmented), which is performed manually, rarely semiautomatically, and without further detailed quantitative analysis of mitochondria^16,26–29^. As a result, important insights into understanding of the observed phenotypes may be missed. Therefore, there is a need for an easy-to-use, fully automated approach for evaluating mitochondrial morphology that can precisely segment mitochondria and quantitatively describe both mitochondrial size and shape using the conventional wide-field fluorescence images.

Here, we present the retrained MitoSegNet (MitoS_yeast) deep learning model for yeast mitochondria, which was found to outperform and provide more accurate segmentation of both tubular and spherical mitochondria in the yeast *Saccharomyces cerevisiae* compared to the standard Global thresholding segmentation available in ImageJ/Fiji. We successfully used the retrained model to segment mitochondria in a strain lacking the *MMI1* gene encoding the Mmi1 protein, which is a yeast ortholog of the human “Translationally Controlled Tumor Protein” (TCTP). Mmi1 affects proteasome activity^30^, autophagy^31^ and relocalizes from the cytosol to stress granules, the nucleus^30^, and mitochondria^32^ under stress conditions. However, the molecular mechanism underlying the Mmi1-specific effect on mitochondria is poorly understood.

To quantify the mitochondrial morphology in the *mmi1Δ* and wild-type strains after the MitoS_yeast-mediated segmentation, we used analysis tools available in the ImageJ/Fiji and MitoA analysis methods available in the MitoSegNet analysis toolbox. We obtained a wide range of morphological parameters detailing various aspects of the mitochondrial fragments, such as their size, shape, length, thickness, circularity, compactness, filamentousness, and a branching degree. Our results showed that the *mmi1Δ* cells have larger, longer, thicker, and more branched tubular mitochondria upon oxidative stress than the wild-type cells. Furthermore, evaluation of mitochondrial mass revealed its preservation in the *mmi1Δ* cells under oxidative stress, in contrast to the severe reduction observed in the wild-type cells. This suggests that the mitochondria in the in the *mmi1Δ* strain fail to be degraded during oxidative stress. Illustratively, this conclusion would not be possible without proper quantification of the mitochondrial morphological parameters. Furthermore, we showed that the deep learning model can be retrained to detect mitochondria even from images with high background noise. This demonstrates the high plasticity and broad applicability of the method. The retrained deep learning models for the yeast mitochondrial segmentation and all macros used to run the analyses are now available to other users. To our knowledge, this is the first tool containing a deep learning model for the yeast mitochondrial segmentation.

## Results

### The MitoSegNet deep learning model, retrained on the yeast *Saccharomyces cerevisiae*, segments mitochondria more accurately than does the global thresholding segmentation in ImageJ/Fiji

The predominant morphological structure of yeast mitochondria during cell growth manifests as a tubular network. However, upon exposure to hydrogen peroxide, tubular mitochondria form spherical structures^2,16^, similar to the mitochondria of higher eukaryotes^33,34^. We searched for an optimal method that would allow the segmentation of both tubular and spherical (fragmented) mitochondria, as the most accurate mitochondrial segmentation is a critical prerequisite for the subsequent morphological analysis of the detected objects.

Here, we compared two segmentation methods: (1) Global-thresholding-based segmentation available in ImageJ/Fiji, which was run by our macro 1 (MITO_MULTI_GLOBAL_THRESHOLDING.ijm, see Methods for details), and (2) MitoSegNet segmentation (MitoS), a deep learning neural network model designed for mammalian mitochondria^20^, which we retrained for the yeast cells and named MitoS_yeast (see Methods for details). The retraining step was necessary because the original neural network did not correctly segment the yeast mitochondria labeled with mitochondria-targeted green fluorescent protein (mtGFP)^35^. Indeed, mainly false negative but also false positive segmentations were detected by the original neural network when compared to the manually labeled ground truth (see Supplementary Fig. S1 and Methods). The false negative segmentation represents true pixels that are misclassified as background, and the false positive segmentation represents background pixels that are recognized as true pixels.

**Fig. 1:**
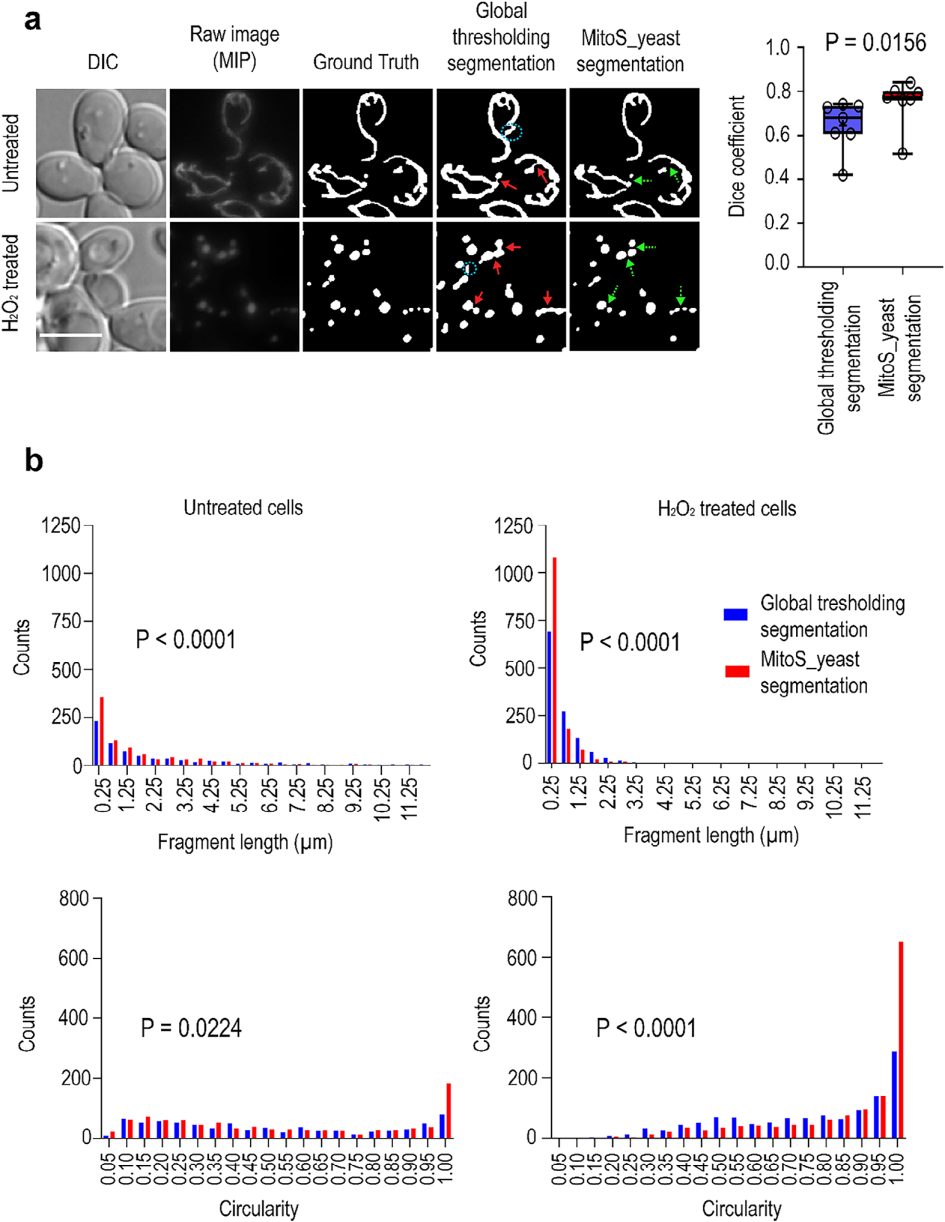
The retrained deep learning segmentation model MitoSegNet (MitoS_yeast) is a suitable tool for detecting tubular and spherical mitochondria in yeast. (**a**) Wide-field fluorescence microscopy image of wild-type yeast mitochondria labeled with mtGFP without any postprocessing brightness adjustment. The raw images represent the maximum intensity projections (MIP) of serial optical sections (z-stacks) taken every 250 nm. Both untreated and treated cells (3 mM H_2_O_2_, 2 h) are shown. Each fluorescence image was supplemented by a corresponding differential interference contrast (DIC) image, Ground Truth, Global thresholding segmentation image and MitoS_yeast segmentation image. The blue dashed circles indicate additional artificial “ghost” segments detected by Global thresholding segmentation. Red arrows indicate areas where worse segmentation occurred, and green arrows with dashed lines indicate areas of better segmentation. Scale bar: 5 μm. The results of the dice coefficients are presented as the means per image (*n* = 7) and are organized in box plots with maximum and minimum whiskers, all calculated values (circles), means (+), and medians (lines). The nonparametric Wilcoxon matched-pairs signed rank test was used to test the difference between the methods. In total, 381 cells from 7 images were analyzed: 182 cells were untreated, while 199 cells were treated with H_2_O_2_. (**b**) Distributions of fragment length and circularity after segmentation by Global thresholding segmentation (blue columns) and MitoS_yeast segmentation (red columns). The results in the histograms represent the value per fragment. In total, more than 300 cells from two independent experiments were analyzed for each treatment. The nonparametric Mann–Whitney U test was used to test the difference between the segmentation methods.

To compare the Global-thresholding-based segmentation and the neural network model MitoS_yeast retrained for yeast cells, both tubular (untreated) and spherical (H_2_O_2_-treated) mitochondria were analyzed by standard wide-field fluorescence microscopy. For segmentation maximum intensity projections (MIP) of z-stacks were used. Global thresholding segmentation often failed to separate isolated fragments (Fig. 1a, red arrows), and additional (ghost) fragments were detected (Fig. 1a, blue circles with dashed lines). In contrast, the MitoS model detected separated mitochondrial fragments more accurately, did not show additional fragments, and did not artificially fragment the tubular mitochondrial network (Fig. 1a, green arrows with dashed lines). To quantitatively compare the accuracy of the segmentation methods, we calculated the Dice coefficient (see Methods for details). The Dice coefficient quantifies the overlap between the predicted segmentation and the ground truth, penalizing both false negatives and false positives. Its value ranges from zero (indicating no overlap) to one (indicating 100% overlap). As shown in Fig. 1a, the MitoS_yeast deep learning neural model significantly outperformed the Global thresholding segmentation (*P* = 0.0156). Further, our observations suggested that the Global thresholding segmentation method artificially detects longer and less circular mitochondria than does the MitoS model. To test this hypothesis, we calculated the fragment length and the circularity of the segmented mitochondria. The circularity describes the fragment’s shape and ranges from 0 to 1 (0.0 indicates a line segment shape, and 1.0 indicates a perfect circle). To calculate the fragment length and circularity, we used tools available in ImageJ/Fiji, which were run by our macro 2 (MITO_CELL_BASED_ANALYSIS.ijm; see Methods for details). As shown in Fig. 1b, significantly longer and less circular fragments were indeed detected by the Global thresholding segmentation method compared to the deep learning MitoS segmentation model. Taken together, these results demonstrate that the retrained deep learning MitoS_yeast model is the more suitable method for the segmentation of yeast mitochondria. Not only does it segment both tubular and spherical mitochondria more accurately using conventional wide-field fluorescence images, but it is also a fully automated, and easy-to-use tool. Moreover, if necessary, the deep learning model can be further trained to detect mitochondria of different yeast species and under different growth conditions. The MitoS_yeast segmentation model, including the macros used for Global thresholding-based segmentation and for the morphological analysis are now freely available to other users (see Methods for details).

### Cells lacking the *MMI1* gene form larger, longer, thicker, more tubular, less compact, and more filamentous and branched mitochondria when exposed to oxidative stress compared to wild-type cells

**Fig. 2:**
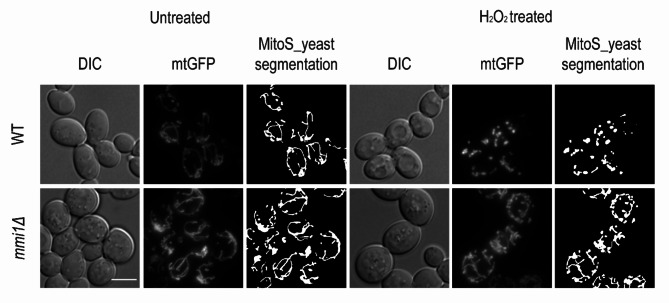
Upon oxidative stress, the mitochondria of the wild-type strain appeared to be more fragmented than the mitochondria of the *mmi1Δ* strain. Late exponentially growing wild-type and *mmi1Δ S. cerevisiae* cells (OD_600_ ≈ 3) possessing mitochondrial GFP (mtGFP) were treated with 3 mM H_2_O_2_ for 2 h. Live cells were examined by fluorescence microscopy, and serial optical sections of the cells in the GFP channel were taken every 250 nm. Fluorescence images represent the maximum intensity projections (MIP) of all optical sections without any postprocessing brightness adjustment. Each fluorescence image was supplemented by a corresponding differential interference contrast (DIC) image and MitoS_yeast segmentation image. Scale bar: 5 μm.

Next, we wished to evaluate mitochondrial morphology in the wild-type and *mmi1Δ* strains harboring a deletion in the *MMI1* gene, which encodes the Mmi1 protein. The Mmi1 protein was shown to bind to mitochondria under oxidative stress in late exponentially growing yeast cells^32^, but its effect on their morphology is poorly understood. As shown in Fig. 2, fluorescence microscopy suggested that the *mmi1Δ* strain had a less fragmented mitochondrial network compared to wild-type strain when exposed to 3 mM H_2_O_2_ for 2 h. However, we could not draw any further conclusions without a detailed quantitative analysis of the morphological parameters.

**Fig. 3:**
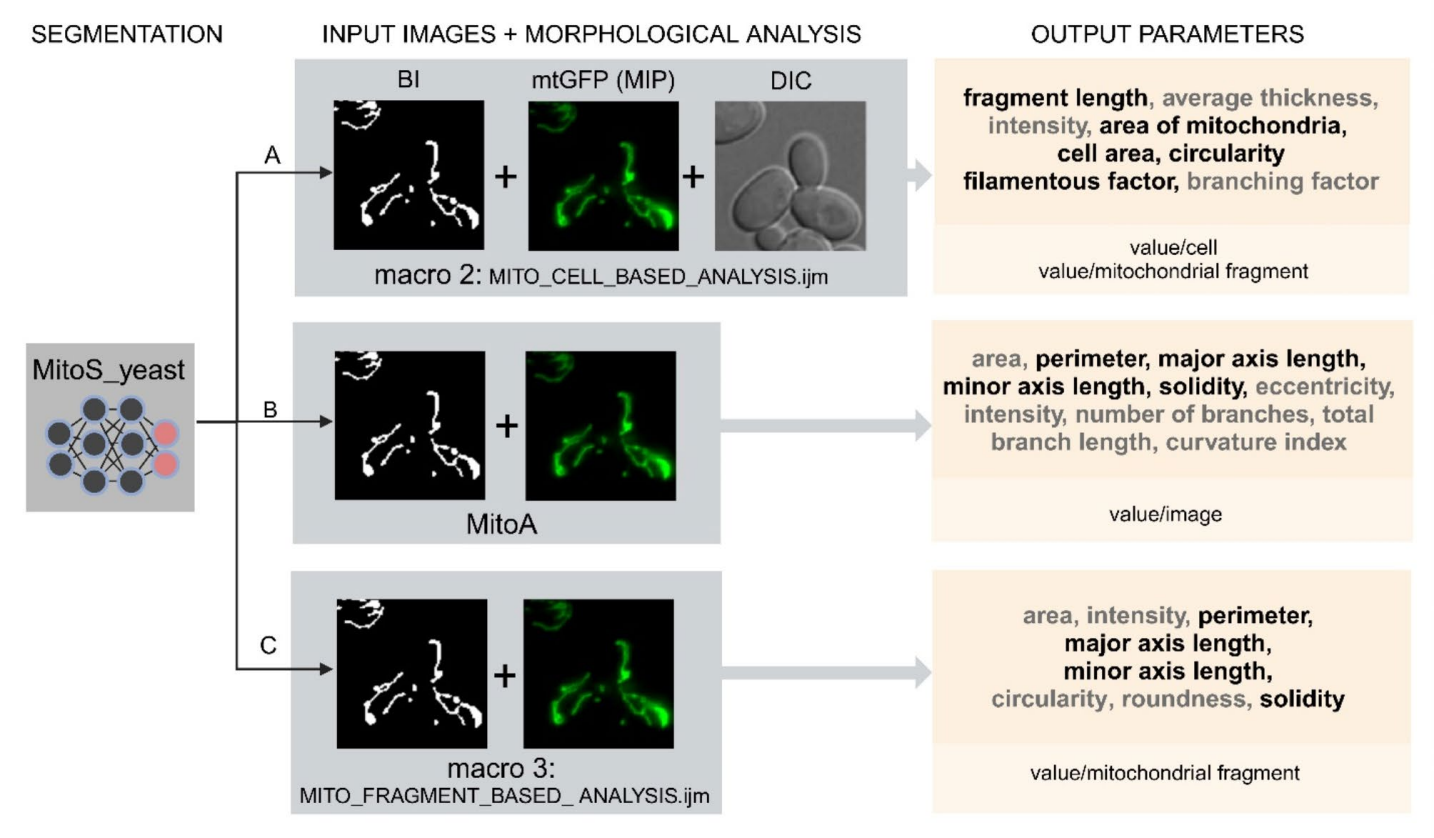
Three different approaches used to quantify mitochondrial morphology. The approach A uses 3 types of images: binary image (BI) of segmented mitochondria, fluorescence maximum intensity projection (MIP) image and differential interference contrast (DIC) image and ImageJ/Fiji analyses run by macro 2. Output parameters can be expressed as values/mitochondrial fragment or values/cell. The output parameters in black are those used for consequent quantitative analysis of mitochondria in the *mmi1Δ* and wild-type strains. The parameters in gray are other parameters that can be calculated by the analysis. The approach B uses BI of segmented mitochondria and fluorescence MIP image as input images for MitoA analysis that calculates output parameters as values/image. The approach C uses BI of segmented mitochondria and fluorescence MIP image and ImageJ/Fiji analysis run by macro 3 that calculates output parameters as value/mitochondrial fragment. mtGFP-mitochondrial target GFP, MitoS_yeast (segmentation by the deep learning model MitoSegNet retrained for yeast cells), and MitoA (morphological analysis in the MitoSegNet analysis toolbox). The figure was created with BioRender.com.

We used our preferred MitoS_yeast model to segment mitochondria in the wild-type and *mmi1Δ* cells under control versus oxidative stress conditions. To quantitatively analyze the morphological parameters of segmented mitochondria in the *mmi1Δ* and wild-type strains, we used 3 different approaches, as summarized in Fig. 3. First, we performed analysis by our previously used macro 2 (MITO_CELL_BASED_ANALYSIS.ijm), which applies plugins such as local thickness, skeletonization, particle analysis, Cellpose^36^ and a few others available in ImageJ/Fiji. Second, we used the MitoA analysis freely available in the MitoSegNet analysis toolbox^20^, and third, we used our custom-written macro 3 (MITO_FRAGMENT_BASED_ANALYSIS.ijm), which exploits analyses provided by the particle analysis plugin in ImageJ/Fiji (see Methods for details).

For the first analysis performed by the macro 2, binary image (BI) of segmented mitochondria, fluorescence maximum intensity projection (MIP) image of z-stacks and differential interference contrast (DIC) image were used as input images. As output results, both the values/cell and values/mitochondrial fragment were obtained (Fig. 3, line A). This first analysis calculated a wide range of parameters, from which we selected basic morphological characteristics concerning the size and shape of mitochondria, such as the ratio of the mitochondrial area to the total cell area, the mitochondrial fragment length, and the circularity of mitochondria. In addition, we also calculated the filamentous factor (FF) that describes the structure and branching of the mitochondrial network. The higher the FF is, the more filamentous and branched the mitochondrial network is. The FF also detects individual filaments, in contrast to the so-called branching factor^37^, which only detects branching (see Supplementary Methods and Supplementary Table S2 for details).

**Fig. 4:**
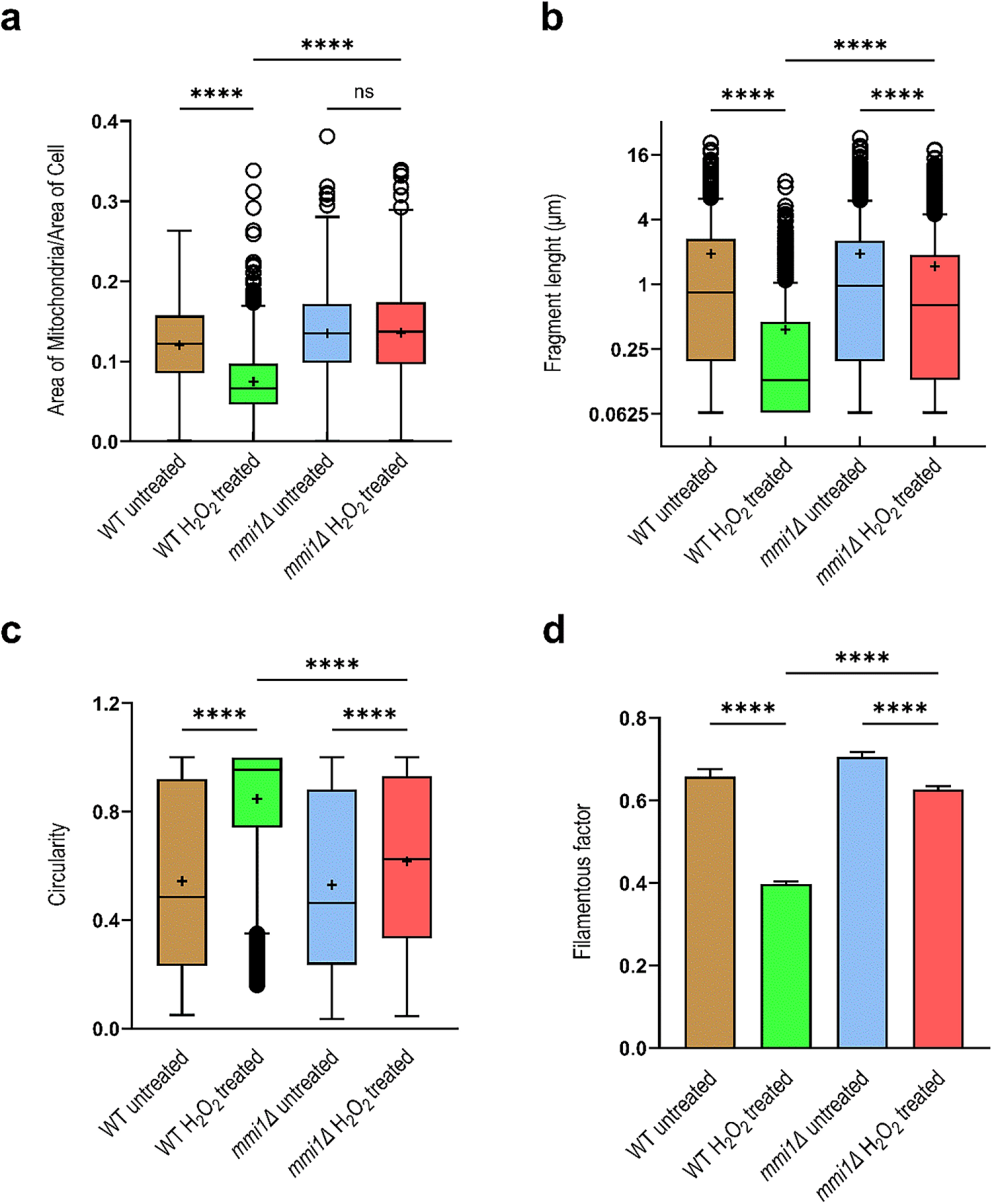

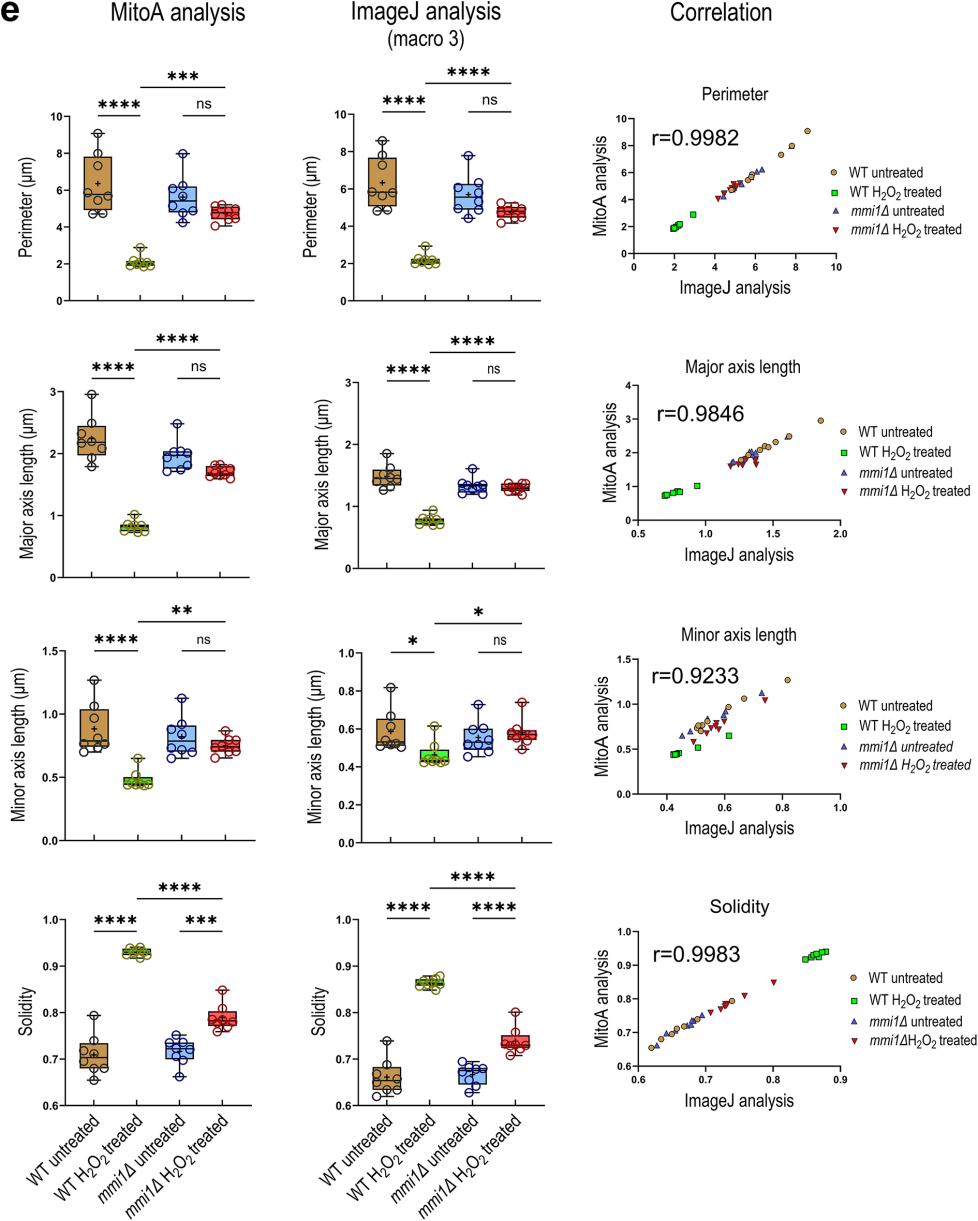
Quantitative evaluation of mitochondrial morphology revealed a striking difference between wild-type and *mmi1Δ* mutant cells after oxidative stress. Mitochondria as shown in Fig. 2 and segmented by the MitoS_yeast deep learning model were quantitatively analyzed by ImageJ/Fiji analyses performed by macro 2. The results of output parameters: **a**) mitochondrial area to the cell area, **b**) fragment length, and **c**) circularity are presented as box plots with Tukey whiskers, means (+), medians (lines), and circles as outliers. The y-axis for fragment length is on the log2 scale. **d**) The results of filamentous factor are presented as the means ± SEs. The nonparametric Kruskal‒Wallis test, followed by Dunn´s multiple comparison test, was used to test the differences among the samples. The data of more than 300 cells from two independent experiments were analyzed for each treatment. **e**) Comparison of the MitoA analysis with the ImageJ/Fiji analysis run by macro 3. The parameters such as perimeter, major axis length, minor axis length, and solidity were tested. The results are presented as the means per figure (*n* = 8) and are organized in box plots with maximum and minimum whiskers, all calculated values (circles), means (+), and medians (lines). One-way ANOVA was used to test the difference between the samples. The correlation analysis of the results of the MitoA analysis and the ImageJ/Fiji analysis (macro 3) is shown with the calculated Pearson´s correlation coefficient (r). ***** *P* ≤ 0.0001, *** *P* ≤ 0.001, ** *P* ≤ 0.01, * *P* ≤ 0.05, ns, not significant.

As shown in Fig. 4a, after the H_2_O_2_ treatment, mitochondria of the wild-type strain showed a drastic decrease in the mitochondrial area-to-cell area ratio in contrast to normal conditions. However, no difference was detected between the treated and untreated *mmi1Δ* cells. Furthermore, the peroxide treatment of wild-type cells resulted in much shorter (Fig. 4b) and more circular mitochondria (Fig. 4c), as well as a highly decreased FF (Fig. 4d). Mitochondria in the *mmi1Δ* strain upon the H_2_O_2_ treatment, also exhibited notably shorter length (Fig. 4b) and increased circularity (Fig. 4c), with a reduced FF (Fig. 4d) compared to normal conditions. However, these alterations were less pronounced than those observed in wild-type cells. These results clearly demonstrate that peroxide treatment dramatically affects mitochondrial mass, length, and shape in wild-type cells, partially in the *MMI1*-dependent manner.

Next, we performed the second analysis using the MitoA analysis^20^, which is operated by a user-friendly stand-alone program. The MitoA analysis tool uses binary image (BI) of segmented mitochondria and fluorescence maximum intensity projection (MIP) image of z-stacks as input images, and the output results are presented as values/image (Fig. 3, line B). It also calculates a wide range of morphological parameters, but, in addition, it allows a multi-correlation analysis of these parameters. We monitored several morphological parameters of mitochondria, such as the perimeter, major axis length (indicating the longest possible distance between two points on the ellipse attached to each mitochondrial fragment), minor axis length (indicating the shortest possible distance between two points on the ellipse attached to each mitochondrial fragment), and solidity (see Supplementary Fig. S2). The solidity parameter describes the ratio of the area of the particle relative to the area of the convex hull of the particle. The convex hull is the smallest convex shape that can enclose the object completely. Solidity ranges from 0 to 1 and its value describes how compact (packed) the objects is. More compact particles have a higher solidity value, while particles with protruding filaments have a lower solidity value (see Supplementary Fig. S2). First, we performed a multi-correlation analysis of these parameters. As shown in Supplementary Fig. S3a, the correlation analysis of untreated cells demonstrated a random distribution of values obtained from wild-type and *mmi1Δ* cells. However, the analysis of H_2_O_2_-treated cells (Supplementary Fig. S3b) demonstrated a clear separation and clustering of the respective wild-type and *mmi1Δ* values, indicating a different effect of hydrogen peroxide on the tested parameters in wild-type and *mmi1Δ* cells. Next, we compared parameters obtained with MitoA with the same parameters obtained by the third independent morphological analysis available in ImageJ/Fiji performed with our custom macro 3. The input images for this analysis were also binary image (BI) of segmented mitochondria and fluorescence maximum intensity projection (MIP) image of z-stacks, but the output results are the values/mitochondrial fragment (Fig. 3, line C). To compare the results of macro 3 analysis with the results of the MitoA analysis, we recalculated them to values/image. As shown in Fig. 4e, the MitoA and macro 3 analysis were perfectly comparable with high statistical significance, as indicated by high Pearson’s correlation coefficients. Both analyses clearly indicated that H_2_O_2_ treatment decreased the perimeter, major axis length, and minor axis length and increased the solidity of mitochondria in wild-type cells compared to *mmi1Δ* cells. Taken together, all the morphological parameters tested clearly revealed severe collapse of the mitochondrial network and a reduction in mitochondrial mass in the wild-type cells after H_2_O_2_ treatment, whereas the mitochondrial network in the *mmi1Δ* cells remained much more preserved. This suggests that oxidative stress leads to the degradation of mitochondria in wild-type cells, which might be mediated by the Mmi1 protein.

### The MitoS deep learning model can be trained to segment complex mitochondrial networks in images with high background noise

So far, we have demonstrated that the neural network MitoS_yeast can be successfully used to segment tubular and fragmented spherical mitochondria in control and hydrogen peroxide treated wild-type and *mmi1Δ* cells. Next, we wanted to test whether the model can segment another type of mitochondrial network. For this purpose, the prominent yeast fission *dnm1Δ* mutant was chosen, which leads to hyper-fused mitochondria^38^. Yeast cells were grown in medium with 3% galactose, mitochondria were labeled by aconitase-GFP and cells were examined by standard wide-field fluorescence microscopy. To increase the resolution of complicated mitochondrial network, deconvolution of optical sections was performed (see details in Methods) before MIP images were created.

As shown in Supplementary Fig. S4, only a small number of cells contained labeled mitochondria. After contrast enhancement of MIP images aconitase-GFP unlabeled cells and dead cells showed autofluorescence signal (indicated by yellow stars) and artificial spots were also detected in background (indicated by red arrows). It is important to note that this background noise could not simply be subtracted from the image as background without losing part of the mitochondrial signal. When the retrained MitoS_yeast neural network was used to segment the mitochondria, it also segmented some autofluorescence from unlabeled cells and the artificial spots in addition to the mitochondria. This result is understandable because MitoS_yeast was trained to detect both tubular and fragmented spherical mitochondria, and fragmented spherical mitochondria are the most similar to artificially detected structures. Furthermore, the segmentation of mitochondria, especially those forming complex web structures in *dnm1Δ* cells, also needed improvement. Hence, we took advantage of the fact that the neural network model can be further trained.

**Fig. 5:**
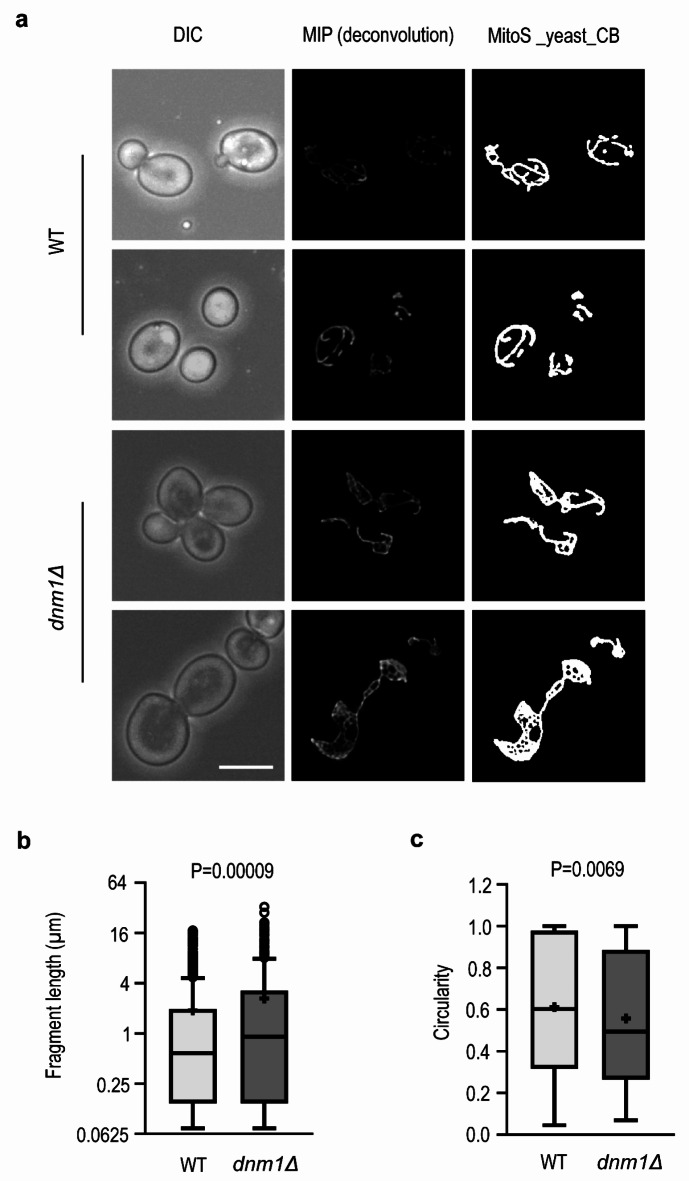
Mitochondria in *dnm1Δ* cells are longer and less circular than mitochondria in WT cells. **a**) Wide-field fluorescence microscopy. The mitochondria were labeled with aconitase-GFP and the fluorescence images represent MIP (maximum intensity projection) images of z-stacks after deconvolution. MitoS-yeast_CB represents segmentation by the deep learning model MitoS trained to detect mitochondria from noisy background. DIC, differential interference contrast. Scale bar: 5 μm. **b**) Fragment length and **c**) circularity are presented as box plots with Tukey whiskers, means (+), medians (lines), and circles as outliers. The y-axis for fragment length is on the log2 scale. More than 150 cells from two different experiments were analyzed in each sample and the Mann–Whitney U test was used to test the difference between WT and *dnm1Δ* mitochondria.

Two independent annotators prepared the training data by hand-labeling ground truth images to train the model for segmenting the tubular and web-like mitochondrial networks while ignoring noise. As shown in Suppl. Fig. S4 and Fig. 5a, the retrained deep learning model largely reduced the presence of noisy structures and improved the segmentation of the mitochondria. We named the model as MitoS_yeast_CB (clean background). We then applied macro 2 to the segmented images and calculated the fragment length and circularity of the fragment. As expected, the results showed higher fragment length and lower circularity of mitochondria in the fission *dnm1Δ* mutant compared to wild-type mitochondria (Fig. 5b, c). Taken together, these results demonstrate the high performance and broad applicability of the neural network model for segmentation of yeast mitochondria.

## Discussion

The segmentation of yeast mitochondria and analysis of their morphological parameters are challenging tasks. Available methods require both super-resolution microscopy images and manual identification of regions of interest, and they offer only a few morphological parameters. A fully automated tool for mitochondrial segmentation from conventional wide-field fluorescence images has been lacking. Here, we compared the Global thresholding segmentation and the retrained deep learning MitoSegNet model (MitoS_yeast) for segmenting tubular and spherical mitochondria in the yeast *Saccharomyces cerevisiae*. We found that the retrained deep learning MitoS_yeast model was more accurate for mitochondrial segmentation than the Global thresholding segmentation (see Fig. 1). Furthermore, while the Global thresholding segmentation requires running macro 1 (MITO_MULTI_GLOBAL_THRESHOLDING.ijm), as well as a manual threshold selection of a suitable threshold by an operator, the deep learning model is a fully automated approach. Moreover, if necessary, the retrained MitoS model can be further trained to achieve better results if mitochondrial segmentation is not satisfactory, e.g., due to alternations in mitochondrial morphology during different growth phases, different media, etc. Indeed, our results demonstrated that the deep learning network can be trained to detect mitochondria even in images with background noise (Supplementary Fig. S4). This capability is particularly useful when working with “sick” strains that contain many dead cells, or when a large number of cells have unlabeled mitochondria that exhibit autofluorescence. And last but not least, the model can also be trained to detect mitochondria in other yeast species or even some organelles, e.g., the tubular endoplasmic reticulum or some spherical structures such as actin patches or stress granules.

For the subsequent morphological analysis of segmented mitochondria, we used analyses in ImageJ/Fiji run by our macro 2 (MITO_CELL_BASED_ANALYSIS.ijm), macro 3 (MITO_FRAGMENT_BASED_ANALYSIS.ijm) and the MitoA analysis available in the MitoSegNet analysis toolbox^20^ (Fig. 3). The macros differ in the input images used, ImageJ/Fiji analyses, output parameters and forms of the output results, which can be expressed as values/cell or values/mitochondrial fragment (macro 2) or values/mitochondrial fragment (macro 3). The MitoA analysis exploits the same input images and calculates nearly the same parameters as macro 3, but the output results are in the form of values/image. We compared the results of the selected parameters calculated by macro 3 with the results of the MitoA analysis, and found both approaches to be suitable and highly comparable. However, there are several details that are worth discussing. As mentioned above, macro 3 allows us to detect morphological parameters per fragment so that a higher number of n values can be obtained. In contrast, the MitoA analysis calculates the values of the tested parameters as the means per image, regardless of the number of mitochondrial fragments in the image. This reduces the total number of tested values (n). Also, the value per image may be highly biased if the number of fragments detected per image is low. On the other hand, MitoA analysis is more user-friendly than macro 3 analysis because it is accompanied by a stand-alone program with convenient dialogs and does not require the execution of a macro in ImageJ/Fiji. In conclusion, we consider both macro 2 and macro 3 as well as the MitoA analysis to be very suitable tools for the quantification of morphological parameters of segmented images. The final selection of an appropriate method requires a thorough consideration of what approach(es) meet the needs of a potential user.

As an example, we applied all these methods to characterize mitochondrial morphology in the wild-type and *mmi1Δ* strains under physiological conditions and upon H_2_O_2_ treatment (Fig. 4). Quantitative morphological analyses revealed that the area, fragment length, perimeter, and major and minor axis lengths of mitochondria are bigger in the *mmi1Δ* strain than in the wild-type strain after H_2_O_2_ treatment. Furthermore, a higher filamentous factor, lower circularity, and solidity values of mitochondrial fragments were detected in the *mmi1Δ* strain under oxidative stress. Taken together, these results clearly demonstrated a preservation of a larger mitochondrial network and a higher mitochondrial mass in the *mmi1Δ* strain under oxidative stress. In contrast, the wild-type strain exhibited pronounced decrease in mitochondrial mass and severely compromised mitochondrial network under oxidative stress. This robust decrease of mitochondrial mass in the wild-type strain under oxidative stress might indicate that one of a cellular role of Mmi1 is to promote degradation of mitochondria under stress conditions. Next, we also applied the MitoS_yeast deep learning model to segment the mitochondria of the *dnm1Δ* fission mutant strain, which forms a hyperfused mitochondrial network resembling a “web”^38^ in contrast to the mitochondrial changes (fragmentation of the mitochondrial network) tested so far. However, the Mito_S model segmented not only mitochondria but also background noise such as dead cells, cell autofluorescence, and artificial circular spots that were present in the images. The segmentation of the mitochondrial network was also unsatisfactory, as the model was not trained to segment hyperfused mitochondria (see Supplementary Fig. S4). However, when an alternative MitoS_yeast_CB deep learning model trained to segment the mitochondrial network of wild-type and *dnm1Δ* strains in the noisy images, was applied, a high improvement in segmentation was achieved (Supplementary Fig. S4 and Fig. 5). Further, calculation of two basic morphological parameters, such as fragment length and circularity by macro 2 demonstrated that the fission mutant *dnm1Δ* possess longer and less circular mitochondrial fragments compared to wild-type strain (Fig. 5b, c). These results are highly consistent with the phenotype of the mutant and demonstrate the tremendous plasticity and wide applicability of the deep learning segmentation.

Taken all together, all tools developed in this work, along with detailed instructions, are freely available on the GitHub repository. We anticipate that this toolbox will be beneficial to researchers who want to describe a yeast mitochondrial morphological phenotype from standard fluorescence images and are searching for an accurate, fast, and user-friendly method. It allows evaluation of differences in mitochondrial morphology that would be difficult or impossible to detect by visual inspection alone, and, hence, a better description of a mitochondrial phenotype. In the future, it will be possible to include mitochondrial morphology as an important parameter of cellular “health/disease”, for which there is an ever growing demand in the scientific community.

## Methods

### Yeast strains and growth conditions

The *Saccharomyces cerevisiae* strains used in this study are listed in Supplementary Table S1. Yeast cells were grown in a shaking Erlenmeyer flask in rich YPD medium (1% w/v yeast extract, 2% w/v peptone, and 2% w/v glucose), in synthetic-defined medium SD (0.17% w/v yeast nitrogen base, 0.5% w/v ammonium sulfate, 2% w/v glucose and amino acids without leucine) or in synthetic-defined medium SDGal without uracil (0.17% w/v yeast nitrogen base, 0.5% w/v ammonium sulfate, 3% w/v galactose, amino acids) at 30 °C.

### H_2_O_2_ treatment

Yeast strains were inoculated into the SD without leucine and grown overnight at 30 °C. Primary cultures were inoculated from the overnight cultures and grown in YPD medium to the late exponential phase (OD_600_ ≈ 3.0). The cells were then treated with 3 mM H_2_O_2_ (Sigma) for 2 h at 30 °C under shaking. After treatment, the cells were examined using fluorescence microscopy.

### Wide-field fluorescence microscopy

The distribution of mitochondria-targeted GFP^35^ was analyzed with a 100x PlanApochromat objective (NA 1.4) using an Olympus IX-71 inverted microscope equipped with a Hamamatsu Orca/ER digital camera and the Olympus Cell R detection and analyzing system (GFP filter block U-MGFPHQ, exc. max. 488, em. max. 507). Serial optical sections (z-stacks) of yeast cells were taken every 250 nm, and a total projection of all sections was used for analysis without further postprocessing contrast adjustment. In addition to images of GFP fluorescence, images of the corresponding yeast cells were obtained via Nomarski differential interference contrast (DIC). Images of wild-type cells and *dnm1Δ* cells labelled with aconitase-GFP were analyzed with 100X CFI Plan Apochromat Lambda D objective (NA 1.45) in Nikon Eclipse Ti2 inverted microscope. The microscope is equipped with LED-FITC-A-2745 A Filter Cube (32 mm) (EX 474/27, DM 495, BA 525/45) and a DS-Qi2 camera with a Monochrome CMOS senser (size: 36.0 × 23.9 mm Effective 16.25 megapixels). The images were taken under the same condition as described above. Deconvolution of z-optical sections was performed in Huygens Professional v. 24.04 software.

### Mitochondrial segmentation and analysis of mitochondrial morphology

Mitochondrial segmentation was performed using two methods: Global thresholding and a deep learning model MitoS from the MitoSegNet project^20^. Global thresholding segmentation is available in ImageJ/Fiji, and to use it, we developed the MITO_MULTI_GLOBAL_THRESHOLDING.ijm macro (makro 1), which preprocesses fluorescence images using background subtraction, grayscale, binary morphology, and appropriate filtering. It also applies Global thresholding with a predefined range of thresholds and eliminates small resulting objects using the particle analyzer in ImageJ/Fiji. An expert then selects the best segmented image with an appropriate threshold. Next, as a second segmentation method, we used a deep learning model. The original MitoSegNet (MitoS) model^20^ was retrained using four of our manually annotated images to improve the segmentation accuracy for yeast mitochondria. Since four images were used for retraining, one epoch was sufficient to obtain a better model named here MitoS_yeast. Using more epochs resulted in over-segmentation of the model. In case of MitoS_yeast_CB (clean background) model that was trained to detect mitochondria from noisy background, 7 epochs/120 image augmentations of the original MitoSegNet model were performed.

To analyze mitochondrial morphology, three input images were available: a fluorescence maximum intensity projection (MIP) image of z-stacks of mitochondria, a differential interference contrast (DIC) image of corresponding yeast cells to associate segmented mitochondria with individual cells, and a binary image (BI) of segmented mitochondria. For image processing of the data in ImageJ/Fiji other macros were developed. The macro MITO_CELL_BASED_ANALYSIS.ijm (macro 2) uses MIP fluorescence images of mitochondria, BIs of segmented mitochondria, and corresponding DIC images. It applies local thickness and skeletonization and determines, e.g., mitochondrial fragment length, thickness, area, intensity, circularity, and parameters to calculate the branching factor^37^ and the filamentous factor. The filamentous factor (FF) is calculated as the sum of all junctions per fragment plus the sum of all branches per fragment divided by the sum of all end-point voxels per fragment detected by skeleton analysis (see Supplementary Methods and Supplementary Table S2 for details). In addition, DIC images are segmented using the Cellpose method to create individual cell masks, and various parameters are measured for each mitochondrial fragment in each yeast cell. The resulting images and numerical results are saved as TIF and CSV files. The macro MITO_FRAGMENT_BASED_ANALYSIS.ijm (macro 3) uses MIP fluorescence images of mitochondria, BIs of segmented mitochondria and analyzes the intensities and morphological features of individual mitochondria (e.g., area, intensity, perimeter, major axis length, minor axis length, circularity, roundness, and solidity) using the particle analysis plugin in ImageJ/Fiji. The output results are saved as CSV files. In addition to the macros, the MitoA analysis available in the MitoSegNet analysis toolbox^20^ was used to evaluate morphological parameters of segmented mitochondria.

### Evaluation of the segmentation methods

The Dice coefficient was used to compare the segmentation accuracy of the global thresholding segmentation with that of the retrained MitoS neural network (MitoS_yeast). Originally established as an ecological measure to quantify associations between species^39^, the Dice coefficient (dc) has become widely used in biomedical image analysis to quantify the similarity between a segmented image and the ground truth image^20,40^. To maintain objectivity in the accuracy evaluation of the methods, the ground truth images were hand-labeled (using Labkit plugin in ImageJ/Fiji) by two annotators who were independent of the annotator who created the training data for the neural network. The Dice coefficient was calculated using Eq. (1), where TP (True Positive) pixels correspond to those present in the expert-generated ground truth. FP (False Positive) segmented pixels represent structures incorrectly included in the segmentation but not present in the ground truth. FN (False Negative) pixels denote structures that are missing in the segmentation compared to the ground truth. TP, FP, FN pixels together with DICE coefficients were evaluated using a Python script that accompanies the original publication about MitoSegNet^20^.

1 $$dc=\frac{2TP\:}{2TP+FP+FN}$$

### Availability of tools for segmentation and quantitative assessment of mitochondrial morphology

The macro for Global thresholding segmentation, the retrained deep learning model MitoS_yeast for yeast mitochondrial segmentation, the MitoS_yeast_CB_deep learning model trained to segment mitochondria from images with high background noise, the macros for morphological analysis of segmented mitochondria, and a detailed protocol for running each macro, including the use of ImageJ/Fiji software, are available on the GitHub repository: https://github.com/LMCF-IMG/Morphology_Yeast_Mitochondria.

### Statistics

All the statistical tests and graphs were performed in GraphPad Prism 10. To test the difference between the dice coefficients between the segmentation methods, first normality Shapiro-Wilk test for low number of values was performed. Since the data had the non-normal (non-Gaussian) distribution and the paired nature, the nonparametric Wilcoxon matched-pairs signed rank test was used. For comparison of morphological parameters among several samples, first normality of the data was tested by the D’Agostino & Pearson normality test. If the data were normally distributed, one-way ANOVA followed by Tukey’s multiple comparison test was used. If the data were not normally distributed, the nonparametric Kruskal‒Wallis test was used, followed by Dunn’s multiple comparison test. If only two groups of non-normally distributed data were compared, the Mann Whitney U test was performed. Pearson’s correlation coefficient was calculated using correlation analysis.

## Electronic supplementary material

Below is the link to the electronic supplementary material.

Supplementary Material 1

## Data Availability

Data generated during the study are available on request from the corresponding author (J.V.). The retrained deep learning models and all created macros are available on the GitHub repository: https://github.com/LMCF-IMG/Morphology_Yeast_Mitochondria.
