# Supplementary material for: A fully automated morphological analysis of yeast mitochondria from wide-field fluorescence images
Source: Sci Rep. 2024 Dec 3;14:30144. doi: 10.1038/s41598-024-81241-0 (PMC11615301; doi:10.1038/s41598-024-81241-0)
Supplement: Supplementary file 1 — Supplementary Material 1 [file 41598_2024_81241_MOESM1_ESM.pdf]

Supplementary Information

A fully automated morphological analysis of yeast mitochondria from wide-field fluorescence images

Jana Vojtová[1,*], Martin Čapek[2,3], Sabrina Willeit[4], Tomáš Groušl[5], Věra Chvalová[5], Eva Kutejová[6], Vladimír Pevala[6], Leoš Shivaya Valášek[1], and Mark Rinnerthaler[4,*]

[1]Laboratory of Regulation of Gene Expression, Institute of Microbiology of the Czech Academy of Sciences; Prague; 14220, Czech Republic

[2]Light Microscopy, Institute of Molecular Genetics of the Czech Academy of Sciences; Prague; 14220, Czech Republic

[3]Laboratory of Biomathematics, Institute of Physiology of the Czech Academy of Sciences; Prague; 14220, Czech Republic

[4]Biosciences and Medical Biology, Paris-Lodron-University Salzburg, 5020, Austria

[5]Laboratory of Cell Signalling, Institute of Microbiology of the Czech Academy of Sciences; Prague; 14220, Czech Republic

[6]Department of Biochemistry and Protein Structure, Institute of Molecular Biology, Slovak Academy of Sciences, Bratislava, 84551, Slovakia

*Corresponding Author: Jana Vojtová, Institute of Microbiology of Czech Academy of Sciences, Vídeňská 1083, Prague,14220, Czech Republic; Phone: +420 241062504; email: jana.vojtova@biomed.cas.cz

*Co-corresponding Author: Mark Rinnerthaler, Department of Biosciences, University of Salzburg, Hellbrunnerstrasse 34, Salzburg, 5020, Austria, email: mark.rinnerthaler@sbg.ac.at

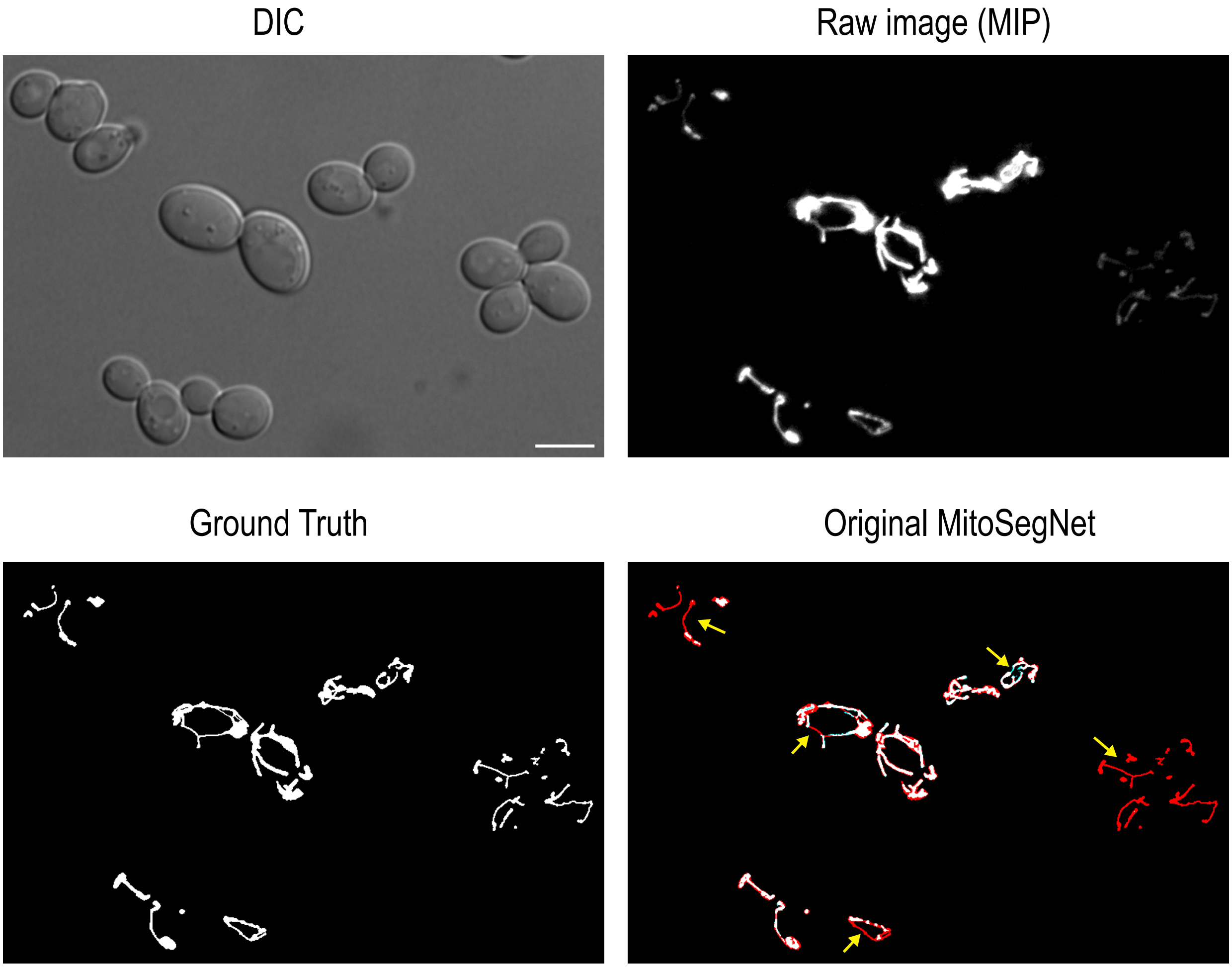

Supplementary Figure S1. Segmentation of tubular mitochondrial network in *S. cerevisiae* by original MitoSegNet neural network. The mitochondria were labeled with mitochondria-targeted green fluorescent protein and analyzed by standard wide-field fluorescence microscopy. The raw fluorescence image represents MIP (maximum intensity projection) image of z-stacks. Its contrast was linearly enhanced (by Brightness/Contrast tool in ImageJ/Fiji) to improve visualization of low intensity structures. The yellow arrows are pointing to the areas with false segmentation. The false negative segmentation is indicated by red color and the false positive segmentation is indicated by blue color. DIC, differential interference contrast. Scale bar: 5 µm.

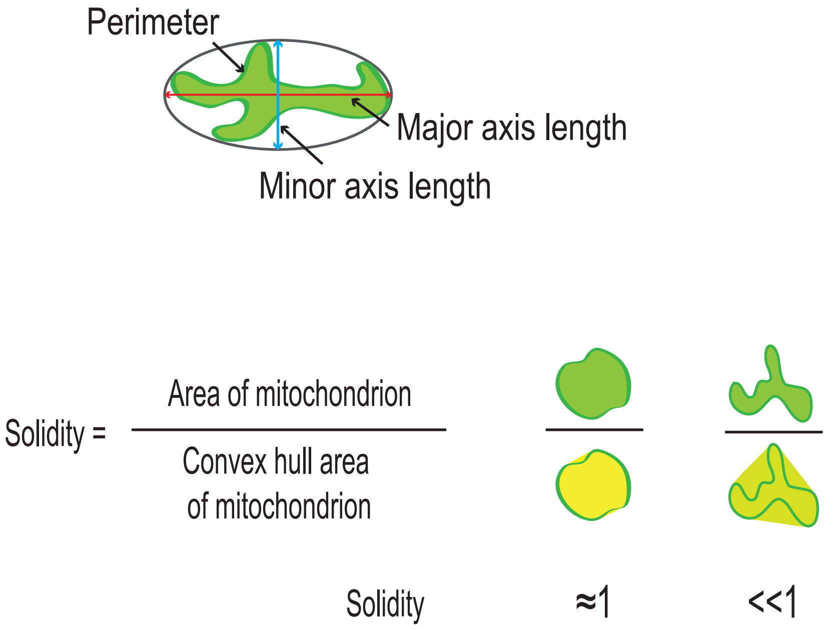

Supplementary Figure S2. Schematic diagrams illustrating the morphological parameters of mitochondria, including perimeter, major axis length, minor axis length, and examples of high and low solidity.

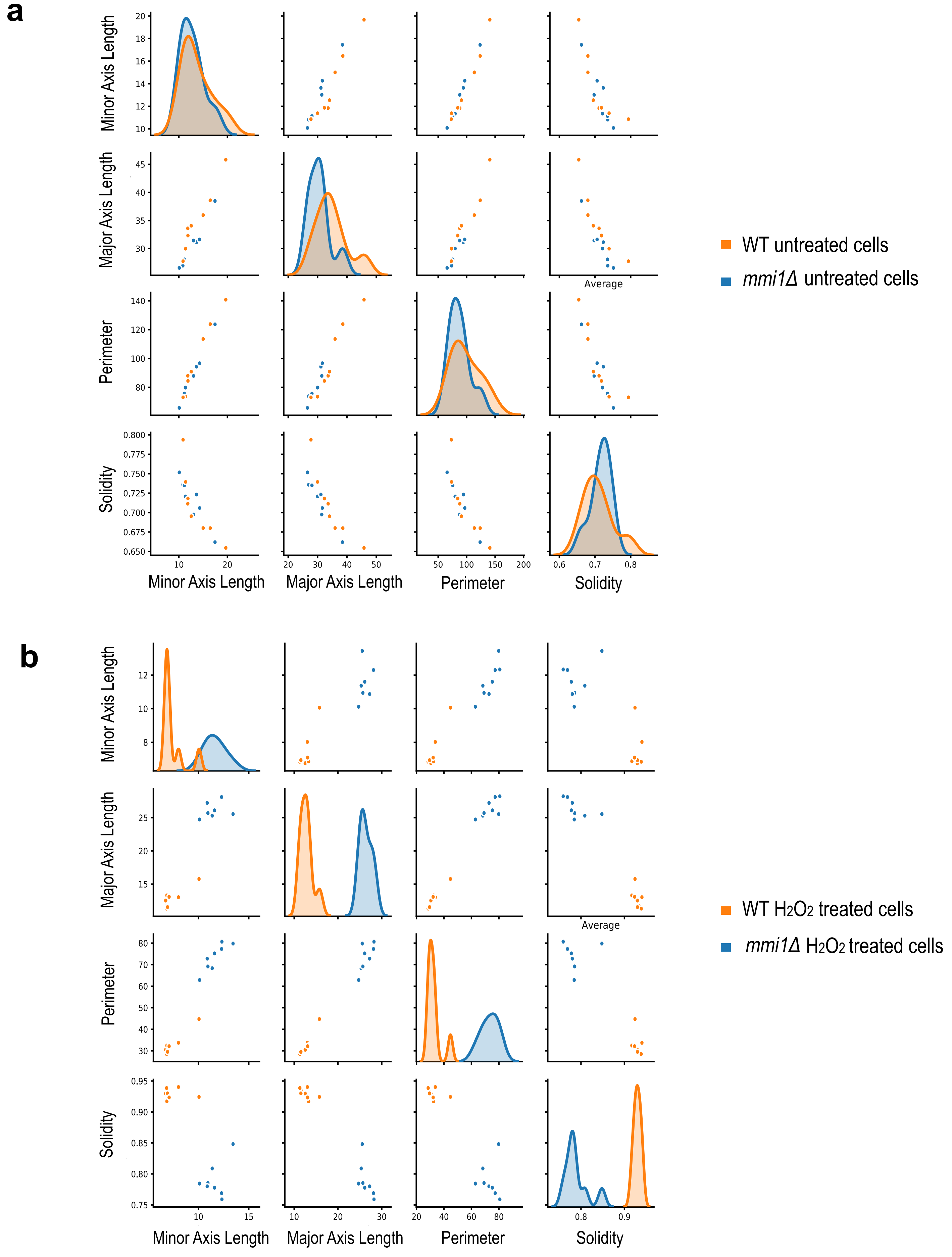
a
Minor Axis Length
Major Axis Length
Perimeter
Solidity
Average
WT untreated cells
mmi1Δ untreated cells
b
Minor Axis Length
Major Axis Length
Perimeter
Solidity
Average
WT H2O2 treated cells
mmi1Δ H2O2 treated cells

Supplementary Figure S3. Correlation analysis of mitochondrial morphological parameters: perimeter, major axis length, minor axis length, and solidity. The wild-type and the mutant *mmi1Δ S. cerevisiae* strains possessing mtGFP were either left **a**) untreated or **b**) treated with 3 mM H_2O_2 for 2 hours. Mitochondria were segmented using the retrained deep learning MitoS_yeast model and analyzed by correlation analysis available in MitoA analysis toolbox. Each spot represents a mean value per image (n=8) from two independent experiments.

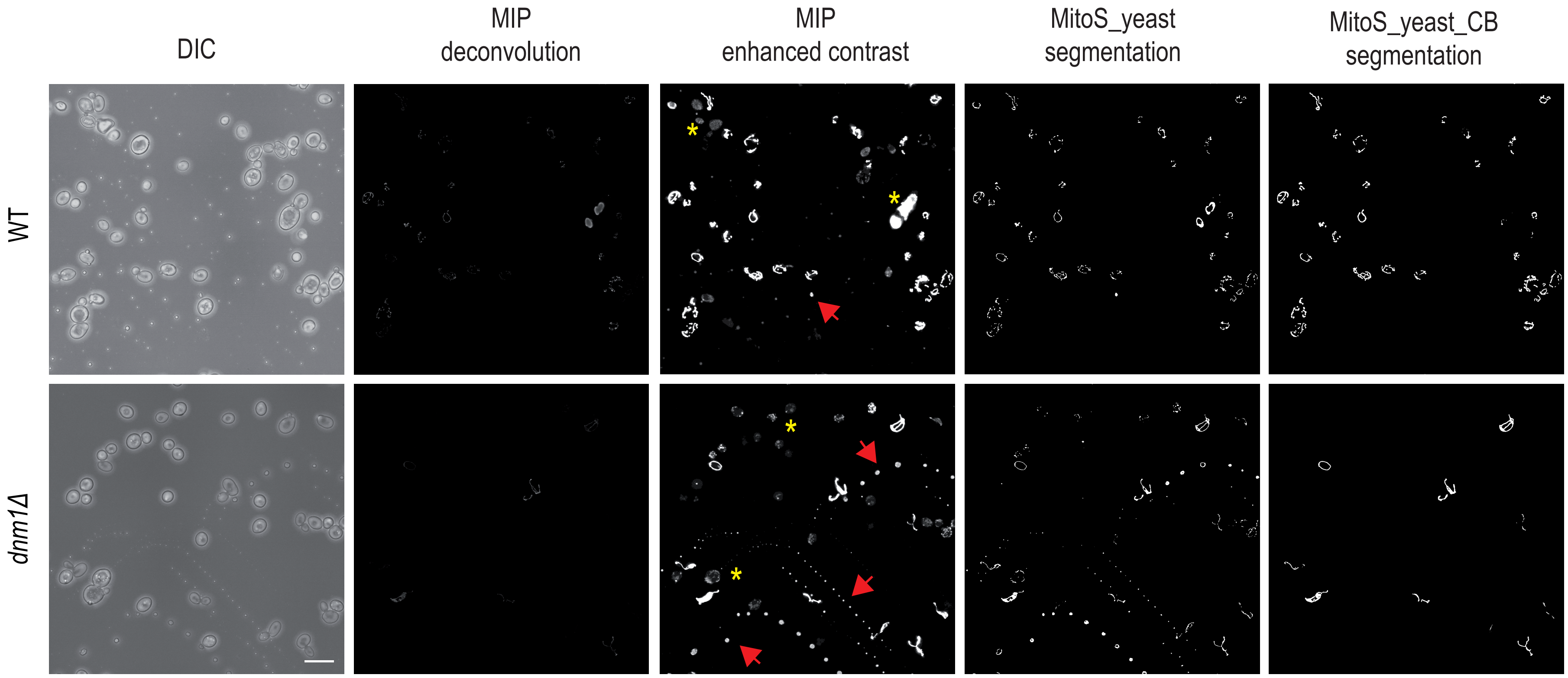
DIC
MIP
deconvolution
MIP
enhanced contrast
MitoS_yeast
segmentation
MitoS_yeast_CB
segmentation
WT
dnm1Δ

Supplementary Figure S4. Segmentation of mitochondrial network in *S. cerevisiae* from images with high noisy background. The mitochondria in WT and *dnm1Δ* cells were labeled with aconitase-GFP and analyzed by standard wide-field fluorescence microscopy. The fluorescence images represent MIP (maximum intensity projection) images of z-stacks after deconvolution. Enhanced contrast MIP are images with linearly enhanced contrast (by Brightness/Contrast tool in ImageJ/Fiji) to improve visualization of low intensity structures. Yellow stars represent dead cells or cells with high autofluorescence, but no aconitase-GFP mitochondria labelling. Red arrows are pointing to background noise (drops). MitoS_yeast represents segmentation by the retrained neural network. MitoS_yeast_CB (clean background) represents segmentation by the MitoS deep learning model trained to detect mitochondria in noisy background. DIC, differential interference contrast. Scale bar: 10 μm.

Supplementary Table S1. *Saccharomyces cerevisiae* strains used in the study.

Strain	Relevant genotype	Source
wild-type	BY4742 *MATα his3Δ1 leu2Δ0 lys2Δ0 ura3Δ0* pXY142-mtGFP [LEU]	M. Rinnerthaler´s laboratory (Salzburg, Austria)
mmi1Δ	BY4742 *MATα his3Δ1 leu2Δ0 lys2Δ0 ura3Δ0 mmi1::natNT2* pXY142-mtGFP [LEU]	M. Rinnerthaler´s laboratory (Salzburg, Austria)
wild-type	BY4741 *MATa his3Δ1 leu2Δ0 met15Δ0 ura3Δ0* pUG35-Aco1-GFP [URA]	M. Rinnerthaler´s laboratory (Salzburg, Austria)
dnm1Δ	BY4741 *MATa his3Δ1 leu2Δ0 met15Δ0 ura3Δ0 dnm1::kanMX4* pUG35-Aco1-GFP [URA]	M. Rinnerthaler´s laboratory (Salzburg, Austria)

Supplementary Table S2. Examples of calculated branching factors and filamentous factors for different mitochondrial fragment shapes.

MITOCHONDRIAL FRAGMENT SHAPE	JUNCTIONS (purple)	END-POINT VOXELS (blue)	BRANCHES (orange)	BRANCHING FACTOR	FILAMENTOUS FACTOR
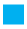	0	1	0	0	0
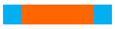	0	2	1	0	$\frac{1}{2}$
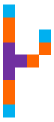	1	3	3	$\frac{1}{3}$	$\frac{4}{3}$

Supplementary Methods: Calculation of branching factors and filamentous factors

The calculation of the branching factor and the filamentous factor was based on the analysis of segmented mitochondrial fragments by the ImageJ/Fiji plugin analyze-skeleton (https://imagej.net/plugins/analyze-skeleton/). The branching factor of the mitochondrial fragment represents the ratio between the number of branch points and the number of end-point voxels, see equation (1).

$$\text{BRANCHING FACTOR} = \frac{\text{BRANCH POINTS (JUNCTIONS)}}{\text{END}-\text{POINTS VOXELS}} \qquad (1)$$

The filamentous factor of the mitochondrial fragment represents the ratio between the number of branch points plus the number of branches and the number of end point voxels, see equation (2). The branching factor describes branching, while the filamentous factor also detects a single filament (see Supplementary Table 2 for details).

$$\text{FILAMENTOUS FACTOR} = \frac{\text{BRANCH POINTS (JUNCTIONS)}+\text{BRANCHES}}{\text{END}-\text{POINTS VOXELS}} \qquad (2)$$
